# Semiquantitative chest computed tomography scoring system to estimate severity in pediatric community-acquired pneumonia

**DOI:** 10.3389/fped.2025.1556349

**Published:** 2025-08-04

**Authors:** Huifeng Fan, Xuehua Xu, Wangchun Dai, Diyuan Yang, Senqiang Zeng, Qiang Zeng, Gen Lu

**Affiliations:** ^1^ Department of Respiratory Infection, Guangzhou Women and Children’s Medical Center, Guangzhou Medical University, Guangzhou, China; ^2^ Department of Imaging, Guangzhou Women and Children’s Medical Center, Guangzhou Medical University, Guangzhou, China

**Keywords:** community-acquired pneumonia, computer tomography, semiquantitative evaluation, score, children

## Abstract

**Introduction:**

A retrospective study assessed the utility of semiquantitative chest Computed tomography (CT) in diagnosing and determining the severity of community acquired pneumonia (CAP) in children.

**Methods:**

The study included pediatric patients with CAP from January 2019 to December 2023. A semiquantitative chest CT scoring system was used based on the extent of lung inflammatory lesions and hydrothorax. The inter-rater reliability between two observers was assessed. The score was then correlated with laboratory results, identified pathogens, and patient prognosis. Receiver operating characteristic (ROC) analysis was employed to evaluate the diagnostic accuracy of the score for identifying severe cases.

**Results:**

The study included 426 patients, with 186 severe cases (43.66%). The intragroup correlation coefficient (ICC) value of the two observers was 0.957 (95% Cl: 0.944-0.970). The results showed a positive correlation between chest CT scores and neutrophil percentage, neutrophil count, and C-reactive protein, procalcitonin, lactate dehydrogenase, and fibrinogen levels and a negative correlation between lymphocyte count and hemoglobin and albumin levels (*P* < 0.001). The patients with coinfection had higher scores than those with single infections (*P* < 0.05). There was a positive relationship between the score and fever duration and length of stay (*P* < 0.001). The area under the curve (AUC) of chest CT score for diagnosing severe cases was 0.805. A score cutoff of >3 had 64.52% sensitivity and 84.17% specificity.

**Conclusion:**

It is practicable and effective that a semiquantitative chest CT scoring system be used for estimating condition and evaluating prognosis of pediatric CAP.

## 1 Introduction

Community-acquired pneumonia (CAP) is an infectious disease driven by both pathogens and host immune responses. Globally, there are 155 million cases of childhood pneumonia annually, resulting in an estimated 0.76 million global deaths and a cause-specific mortality rate of 5.5 cases per 1,000 livebirths in 2015 (1–4). It was reported that a relevant portion of pediatric CAP patients in developing countries, particularly the youngest, became severely ill and even died (5, 6). Consequently, prediction of disease severity is critical to the effective management of individuals with CAP, including the decision to initiate timely treatment and to admit patients to the hospital. Previous studies had worked on the identification of severe CAP based on the clinical presentation and laboratory values and thus facilitated the risk stratification of CAP patients (7, 8). However, radiologic evaluation is an important factor that is often ignored, especially in children. A comprehensive evaluation method could provide a valuable supplement to identify severity, guide therapy, and estimate prognosis in children.

A radiographic evaluation of CAP plays an important role for the following appraisals: (1) diagnosis, (2) evaluation of disease severity, (3) determination of therapeutic effect, (4) presumption of causal organism, and (5) differentiation of contributory diseases other than CAP (9). Computed tomography (CT), on the other hand, is largely reserved for when complications are suspected or where there is difficulty in differentiating CAP from other pathology. Furthermore, CT also plays a crucial role in assessing the severity of pediatric CAP (9). Moreover, the semiquantitative CT scoring method, which has been developed to assess the severity of pulmonary involvement, is clinically significant in evaluating severe cases of adult and pediatric patients with COVID−19 (10, 11). However, it did not clarify the relationship between the extent of inflammation and severity of pediatric patients according to the semiquantitative CT scoring system. The present study aims to determine the relationship between chest semiquantitative CT scores, laboratory findings, and clinical severity in pediatric patients diagnosed with CAP and who underwent CT scanning and to evaluate the significance of the semiquantitative CT scoring system in estimating disease severity.

## 2 Materials and methods

### 2.1 Study population and ethics

We retrospectively enrolled consecutive patients with CAP who were admitted to and treated at a tertiary referral center in China between January 2019 and December 2023. The patients were diagnosed with CAP according to the World Health Organization’s (WHO) evidence-based guidelines for diagnosing pneumonia in children (12). Chest CT was performed according to general indications of CT for CAP (13), including severe or complex pneumonia, pneumonia intractable to antibiotics, recurrent or non-resolving pneumonia, suspected complications, clinical suspicion with normal or questionable radiographic findings, and suspected underlying diseases. Exclusion criteria included known or suspected active tuberculosis, invasive fungal infections, and chronic pulmonary diseases such as asthma, bronchopulmonary dysplasia, idiopathic pulmonary hemosiderosis, interstitial lung disease, and bronchiectasis.

Baseline patient characteristics were recorded at admission, and the laboratory data were collected within 5 days according to the chest CT time to reduce a time-dependent bias.

This prospective, observational study was conducted in accordance with the Strengthening the Reporting of Observational Studies in Epidemiology (STROBE) guidelines. The study protocol was conducted in accordance with the Declaration of Helsinki and approved by the Ethics Committee of Guangzhou Women and Children's Medical Center of Guangzhou Medical University (No. 2021212A01). We obtained all written informed consent from the children's parents or legal guardians for the use of clinical and laboratory data from their medical reports.

### 2.2 Definitions

Severe or non-severe CAP was classified based on clinical features. The diagnosis of severe cases was obtained when the following criteria were fulfilled (14): (1) major criteria, invasive mechanical ventilation; fluid refractory shock; acute need for noninvasive positive pressure ventilation; and hypoxemia requiring fraction of inspired oxygen (FiO_2_) greater than the inspired concentration or flow feasible in the general care area; (2) minor criteria, respiratory rate greater than the WHO classification for age; apnea; increased work of breathing (e.g., retractions, dyspnea, nasal flaring, and grunting); PaO_2_/FiO_2_ ratio <250; multilobar infiltrates; Pediatric Early Warning Score >6; altered mental status; hypotension; presence of effusion; comorbid conditions (e.g., hemoglobin SS disease, immunosuppression, and immunodeficiency); and unexplained metabolic acidosis. Clinicians should consider it to be a severe condition for children with ≥1 major or ≥2 minor criteria.

### 2.3 Microbiological data

Causative pathogens were identified based on the criteria as follows: bacterial culture from sputum, bronchoalveolar lavage (BAL), or pleural fluid samples collected at admission, along with a compatible Gram stain finding using the BACTEC 9120 automated microbial culturing hood system (bioMérieux, France); detection of *Mycoplasma pneumonia* (*M. pneumoniae*) based on a positive immunoglobulin M (IgM) result and an increase in immunoglobulin G (IgG) levels in convalescent vs. initial blood samples by chemiluminescence immunoassay (Pneumoslide IgM ELISA kit, Vircell, Spain) or positivity for *M. pneumoniae* and *Chlamydia pneumoniae* (*C. pneumoniae*) in nasopharyngeal swabs and/or BAL samples by polymerase chain reaction (PCR); detection of respiratory viruses (adenovirus, influenza, parainfluenza virus, rhinovirus, respiratory syncytial virus, bocavirus, metapneumovirus, or enterovirus) in throat or nasopharyngeal swabs and/or BAL samples as determined by TaqMan qPCR.

### 2.4 Chest CT image acquisition

All scans were performed with the patient in the supine position with or without intravenous contrast on a CT scanner. Philips Brilliance 64-slice or Toshiba Aquilion 64-slice spiral CT machine was used for CT examination. Plain scan was performed on each patient, and an enhanced scan was added when necrosis was present. The scan ranged from the apex of the lung to the posterior costophrenic angle. CT scanning parameters: tube voltage, 120 kV, tube current, 25–30 mA; pitch, 1.0; rotation time, 0.5 s; matrix 512 × 512. The acquisition layer thickness is 0.6 or 1 mm. The original data were transferred to the post-processing workstation and reconstructed with the standard algorithm. The thickness of the reconstructed layer was 2 mm, and multiple planar reformation (MPR), volume rendering (VR), and other post-processing were performed. The interval between CT examination and laboratory examination is 1–5 days.

### 2.5 Chest CT visual quantitative evaluation

Chest CT scans were evaluated upon admission. Image analysis and CT scoring were conducted by two experienced radiologists specialized in chest CT imaging, and the final CT scores were determined through consensus. The major CT dimensions, such as infiltration, consolidation, abscess, nodules, atelectasis, cavitation, and hydrothorax, were fully evaluated. In this study, patients’ chest CT scans were graded on a scale of 0–5 to indicate the extent and characteristics of pulmonary infiltration and/or consolidation. The lung lobes were evaluated for the percentage of lung involvement and categorized as follows: 0 (0%), 1 (1%–5%), 2 (6%–25%), 3 (26%–50%), 4 (51%–75% and/or 25% < consolidation ≤ 50%), or 5 (>75% and/or consolidation >50%) (Table 1). The CT score for hydrothorax was determined based on the amount on each side: 1 (mild/moderate amount) and 2 (massive amount); they were delineated based on whether they reached the hilum. The total score is 9. The final score for each case was determined by a third experienced thoracic radiologist.

**Table 1:** Chest CT visual quantitative evaluation of children with community-acquired pneumonia in the study.

Lobar involvement	Score
None	0
Minimal (1%–5%)	1
Mild (6%–25%)	2
Moderate (26%–50%)	3
Severe (51%–75%) and/or 25% < consolidation ≤ 50%	4
Serious (>75%) and/or consolidation >50%	5

CT score of hydrothorax is calculated for each side involvement, as follows: (1) mild/moderate amount; (2) massive amount reaching the hilum as the boundary.

Total score: 9.

### 2.6 Flexible bronchoscopy

Flexible bronchoscopy combined with BAL has become a prevalent intervention for managing CAP associated with pulmonary consolidation or atelectasis. This technique is effective in clearing inflammatory secretions from the airways, alleviating obstructions, and mitigating the detrimental effects of inflammatory responses (15). Flexible bronchoscopy was performed for children with CAP who met the following criteria: extensive pulmonary infiltrates or consolidation with chest CT findings. All bronchoscopies were clinically indicated and were performed under general anesthesia with a flexible bronchoscope. Usually, BAL was carried out in the most-affected area (identified radiologically and/or endoscopically) and using normal sterile saline previously warmed to body temperature (37℃). The protocols for BAL were performed by instilling three or five fractions of the same volume (5–10 ml) into each lobe according to the weight and age of the child (BAL volume to body weight ∼3 ml/kg). The recovery volume of BAL is >40% which was acceptable.

### 2.7 Statistical analysis

Due to the skewed distribution of data, data were expressed as medians [interquartile range (IQR)] for continuous variables or as numbers and percentages for categorical variables. Intragroup correlation coefficient (ICC) was used to test the consistency of chest CT scores of two observers. ICC values <0.4, 0.4–0.75, and >0.75 represent poor, moderate, and good repeatability, respectively. The non-parametric Mann–Whitney *U* rank-sum test was used for two-group analysis of continuous variables, and the Kruskal–Wallis test was used for three-group analysis of continuous variables. All chest CT scores were correlated with laboratory parameters using the Spearman rank correlation. Receiver operating characteristic (ROC) analysis was performed to evaluate the discriminative performance of chest CT score in assessing the severity of the disease. *P* < 0.05 was statistically significant. Data were considered significant at ^∗^*P* < 0 05, ^∗∗^*P* < 0.01, ^∗∗∗^*P* < 0.001, and ^∗∗∗∗^*P* < 0.0001.

Data were analyzed by GraphPad Prism 8.0 and visualized by ggplot2 package of R software (version 3.6.1).

## 3 Results

### 3.1 Demographic and clinical characteristics

**Figure 1:**
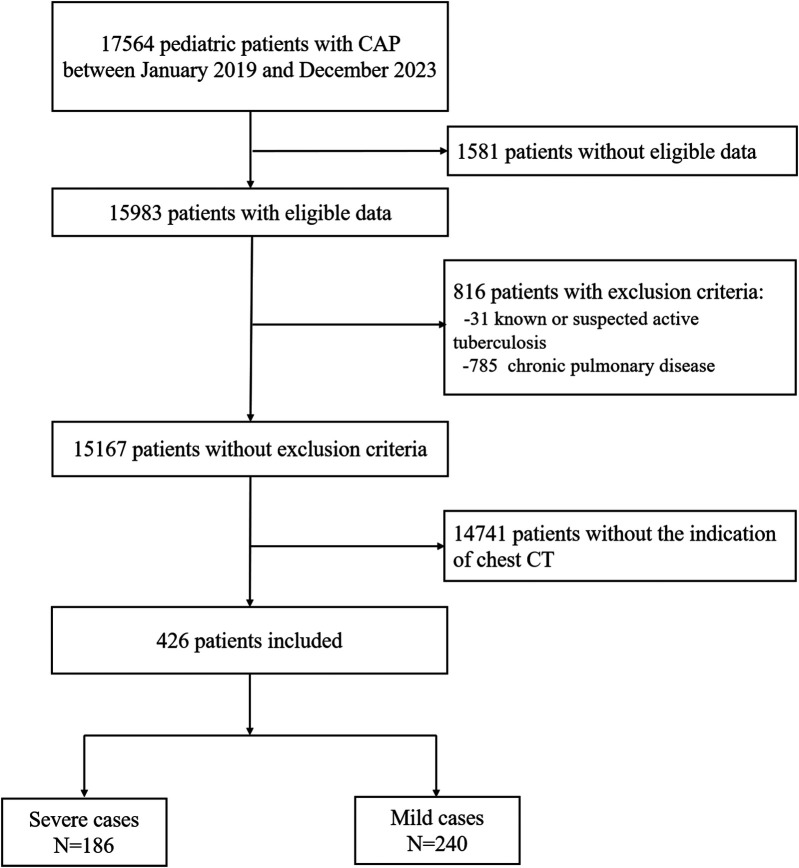
Study flowchart.

A total of 426 patients who underwent chest computed tomography were enrolled in the present study (Figure 1). The demographic and clinical characteristic data for all patients are shown in Table 2. The median age of the patients was 20 months [interquartile range (IQR): 8–48 months], the median weight was 11 kg (IQR: 7.8–15.5 kg), and there were 256 boys (60.01%). Age, weight, and gender showed no correlation with chest CT scores (*P* > 0.05) (Supplementary Figure S1). A total of 186 patients were confirmed as severe cases in this study (43.66%). Thirty-five (8.22%) of the patients had underlying diseases. The most common symptoms were cough (100%), fever (74.18%), and dyspnea (43.66%). All patients were discharged after a median hospitalized period of 10 days (IQR: 7–16days). Eventually, six patients died in this cohort (1.41%).

**Table 2:** Baseline characteristics of children with community-acquired pneumonia in the study.

Variable	Total (*n* = 426)
Demographic	
Age (months), median (IQR)	20 (8–48)
Gender, male (%)	256 (60.09)
Weight (kg), median (IQR)	11 (7.8–15.5)
Severity, no. (%)	186 (43.66)
Underlying diseases, no. (%)	35 (8.22)
Signs and symptoms	
Cough, no. (%)	426 (100.00)
Fever, no. (%)	316 (74.18)
Dyspnea, no. (%)	186 (43.66)
Wheezing, no. (%)	142 (33.33)
Change in level of consciousness, no. (%)	89 (20.89)
Digestive symptoms, no. (%)	74 (17.37)
Laboratory findings^a^	
WBC (×10^9 ^/L) (5–12), median (IQR)	9.2 (6.9–12.5)
NEU(%) (55–65), median (IQR)	45.5 (31–61)
Neutrophil (×10^9 ^/L) (5–12), median (IQR)	3.765 (2.44–6.40)
Hb (g/L) (105–145), median(range)	113 (102–123)
Plt (×10^12 ^/L) (100–300), median (IQR)	381.5 (292–485)
HsCRP (mg/L) (<5), median (IQR)	8.31 (1.18–36.26)
PCT (ng/ml) (<0.25),median (IQR)	0.13 (0.01–0.412)
LDH (U/L) (159–322), median (IQR)	312.5 (257.25–382.75)
ALB (g/L) (40–50),median (IQR)	39.7 (34.73–44.2)
PaO_2_ (kPa) (10.9), median (IQR)	9.95 (6.88–12.54)
PaCO_2_ (kPa) (4.66–5.99), median (IQR)	4.96 (4.45–12.54)
Etiology^b^	
Virus, no. (%)	89 (20.89)
Bacteria, no. (%)	61 (14.32)
Atypical pathogens, no. (%)	87 (20.42)
Coinfection, no. (%)	144 (33.80)
Negative, no. (%)	45 (10.56)
Treatment	
Antibotics, no. (%)	296 (69.48)
Systemic corticosteroid, no. (%)	72 (16.90)
Invasive mechanical ventilation, no. (%)	49 (11.50)
Lobectomy, no. (%)	35 (8.22)
Outcomes	
Length of stay (days), median (IQR)	10 (7–16)
Mortality, no. (%)	6 (1.41)

WBC, white blood count; Hb, hemoglobin; Plt, platelet; HsCRP, high-sensitivity C-reactive protein; LDH, lactate dehydrogenase; ALB, albumin.

^a^ The data of laboratory findings were collected from patients with acute exacerbation.

^b^ The data of etiology were collected in the whole course of the patients.

### 3.2 Chest CT findings

In total, 97.42% (415/426) had CT evidence of pneumonia (Table 3). Pneumonias were in the right upper lobe in 152 cases (35.68%), the right middle lobe in 103 (24.18%), the left lower lobe in 92 (21.60%), the right lower lobe in 63 (14.79%), and the left upper lobe in 56 (13.15%). Among all patients, patchy shadows (305/426, 71.60%) were most common and consolidation more than one lobe (150/426, 35.21%), air bronchograms (93/426, 21.83%), hydrothorax (60/426, 14.08%), inflatable inequality (18/426, 4.23%), and lung abscess (12/426, 2.82%) being the next.

**Table 3:** Chest CT findings of children with community-acquired pneumonia in the study.

Variable	Total (*n* = 426)
CT positive cases, no. (%)	415 (97.42%)
Involving lobes	
Right upper lobe, no. (%)	152 (35.68%)
Right middle lobe, no. (%)	103 (24.18%)
Left lower lobe, no. (%)	92 (21.60%)
Right lower lobe, no. (%)	63 (14.79%)
Left upper lobe, no. (%)	56 (13.15%)
Chest CT signs	
Patchy shadows, no. (%)	305 (71.60)
Consolidation (>1 lobe), no. (%)	150 (35.21)
Air bronchogram, no. (%)	93 (21.83)
Hydrothorax, no. (%)	60 (14.08)
Inflatable inequality, no. (%)	18 (4.23)
Abscess, no. (%)	12 (2.82)
Lymphadenopathy, no. (%)	7 (1.64)
Nodules, no. (%)	3 (0.70)

### 3.3 Interobserver consistency of semiquantitative chest CT score

The consistency test results of CT visual quantitative analysis of two observers showed good repeatability with ICC 0.957 (95% CI: 0.944–0.970) (Supplementary Table S1).

### 3.4 Comparison of semiquantitative chest CT score and laboratory findings

**Figure 2:**
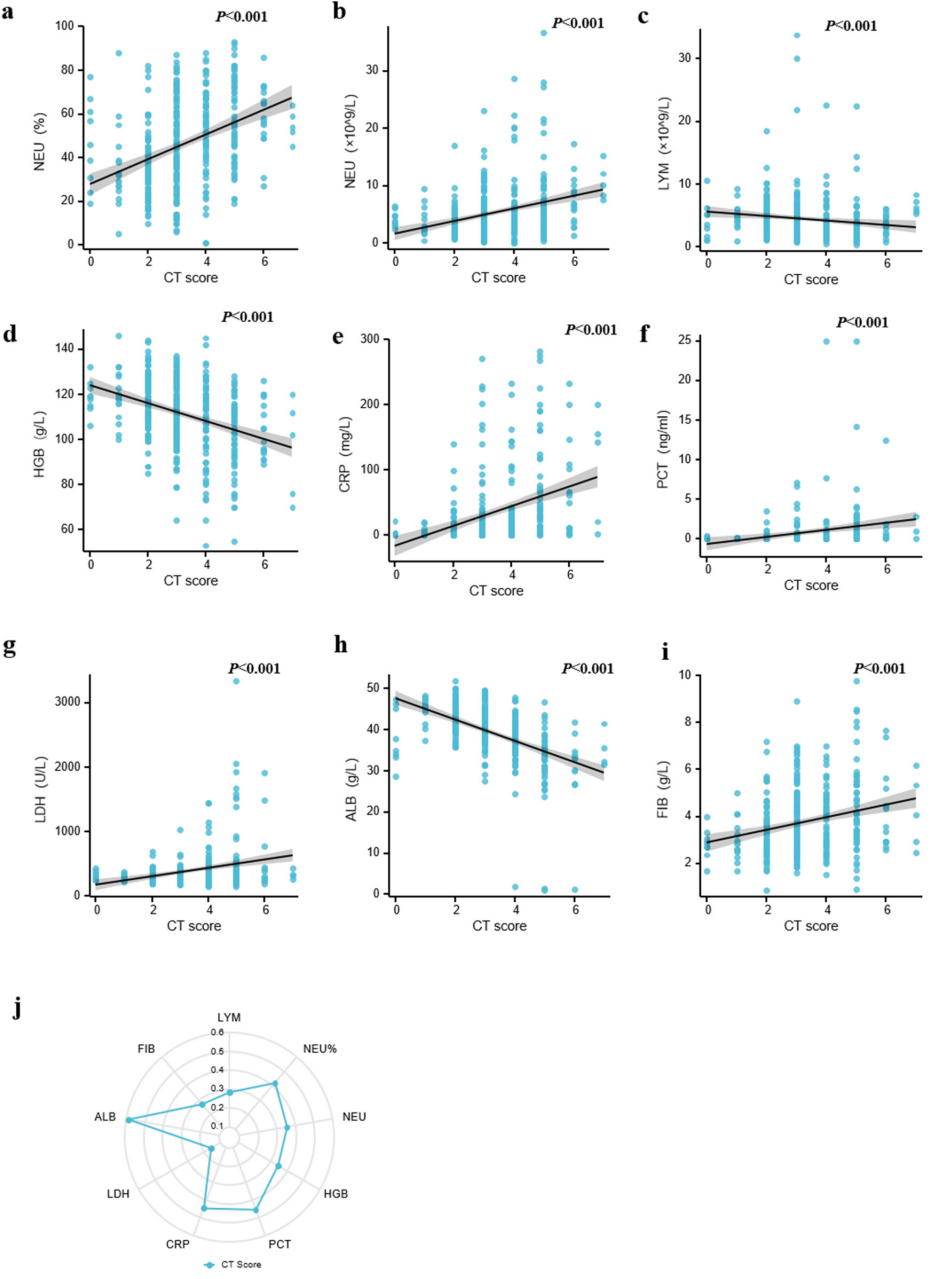
Comparison of semiquantitative chest CT score and laboratory findings. (**a–d)** The correlation between chest CT score and routine blood indexes from children with CAP. **(e**,**f)** The correlation between chest CT score and acute phase reactants from children with CAP. (**g,h)** The correlation between chest CT score and serum biochemical indexes from children with CAP. (**i)** The correlation between chest CT score and FIB from children with CAP. **(j)** The radar map of multiple indexes. NEU, neutrophil; HGB, hemoglobin; LYM, lymphocyte; CRP, C-reactive protein; PCT, procalcitonin; LDH, lactate dehydrogenase; ALB, albumin; FIB, fibrinogen.

To assess the correlation between chest CT scores and laboratory findings, the laboratory data were collected within 5 days of the chest CT scan to minimize any time-dependent bias. There were significant differences in neutrophil percentage (NEU%), neutrophil count (NEU), lymphocyte count (LYM), hemoglobin (HGB), and C-reactive protein (CRP) and procalcitonin (PCT) levels related to chest CT scores (*P* < 0.001) (Figures 2a–f). Lymphocyte count and HGB decreased, whereas NEU%, neutrophil count, and CRP and PCT levels increased with chest CT scores. For serum biochemical indicators, lactate dehydrogenase (LDH) levels increased with chest CT scores, whereas albumin (ALB) decreased with scores (*P* < 0.001) (Figures 2g,h). Moreover, the results showed that fibrinogen (FIB) is positively associated with chest CT scores (Figure 2i**)**. The radar chart showed all biomarkers expressed according to the index of correlation with scores, and ALB showed the strongest associations with scores (Figure 2j**)**. Together, these data suggest that chest CT score is available to indicate the systemic inflammatory response of CAP.

### 3.5 Comparison of semiquantitative chest CT score and pathogens

**Figure 3:**
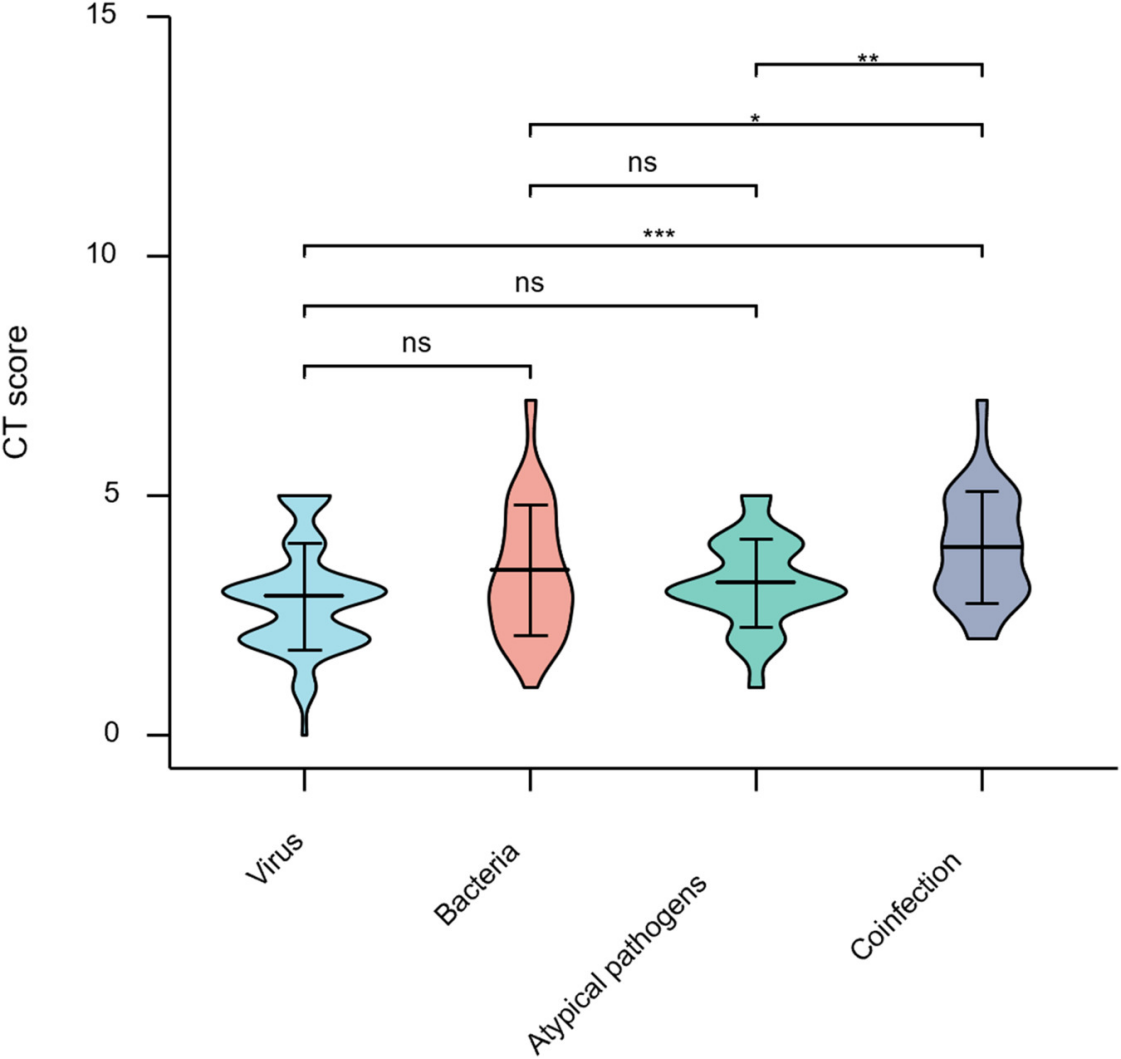
Comparison of semiquantitative chest CT score and pathogens. ns, no significance; *P < 0.05; **P < 0.01; ***P < 0.001; ****P < 0.0001.

To explore the correlation between infectious etiology and chest CT scores, we compared the scores of CAP children infected with viral, bacterial, and atypical organisms and coinfections. There was no statistical difference among the CT scores of viral, bacterial, and atypical organism infections (*P* > 0.05). The CT scores of coinfections were significantly upregulated than those only infected by a single pathogen (*P* < 0.05) (Figure 3; Supplementary Table S2). Thus, we found that coinfection could result in a more serious lung performance in pediatric CAP.

### 3.6 Comparison of semiquantitative chest CT score and severity

**Figure 4:**
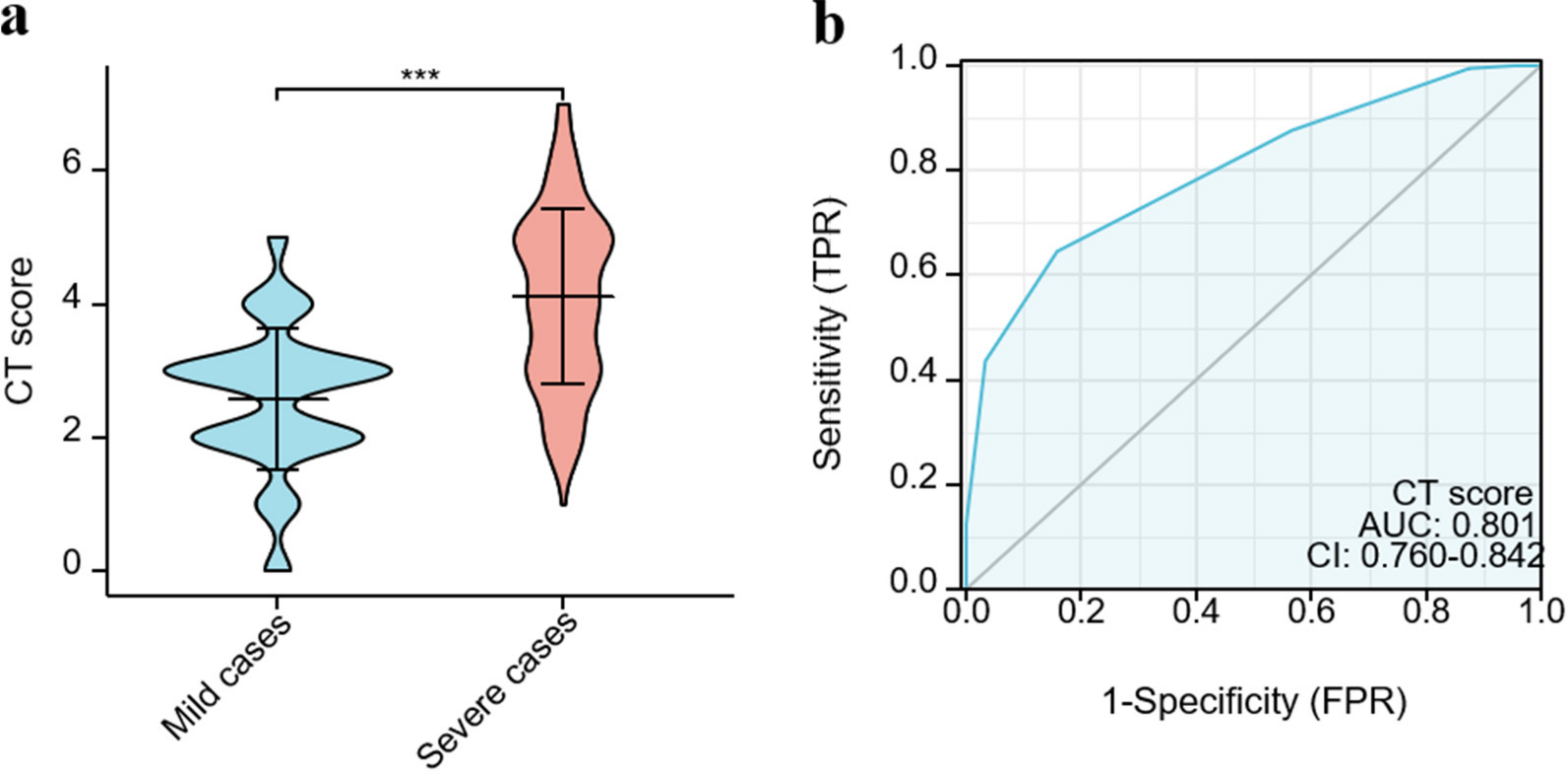
Comparison of semiquantitative chest CT score and severity. **(a)** Comparisons of the chest CT scores of children with CAP according to severity. **(b)** ROC analysis of the ability of chest CT score to predict severe CAP. *P < 0.05; **P < 0.01; ***P < 0.001; ****P < 0.0001.

Next, we compared the chest CT scores in the non-severe vs. severe pediatric CAP group. Chest CT scores were significantly higher in the severe group compared with the non-severe group (*P* < 0.001) (Figure 4a). Our data showed that the diagnostic accuracy of chest CT scores in predicting severe cases was 80.10% (Figure 4b). Based on the ROC analysis, when the cutoff value of chest CT scores was set at >3, the sensitivity and specificity of severe case prediction were approximately 64.52% and 84.17%, respectively.

### 3.7 Comparison of semiquantitative chest CT score and course

**Figure 5:**
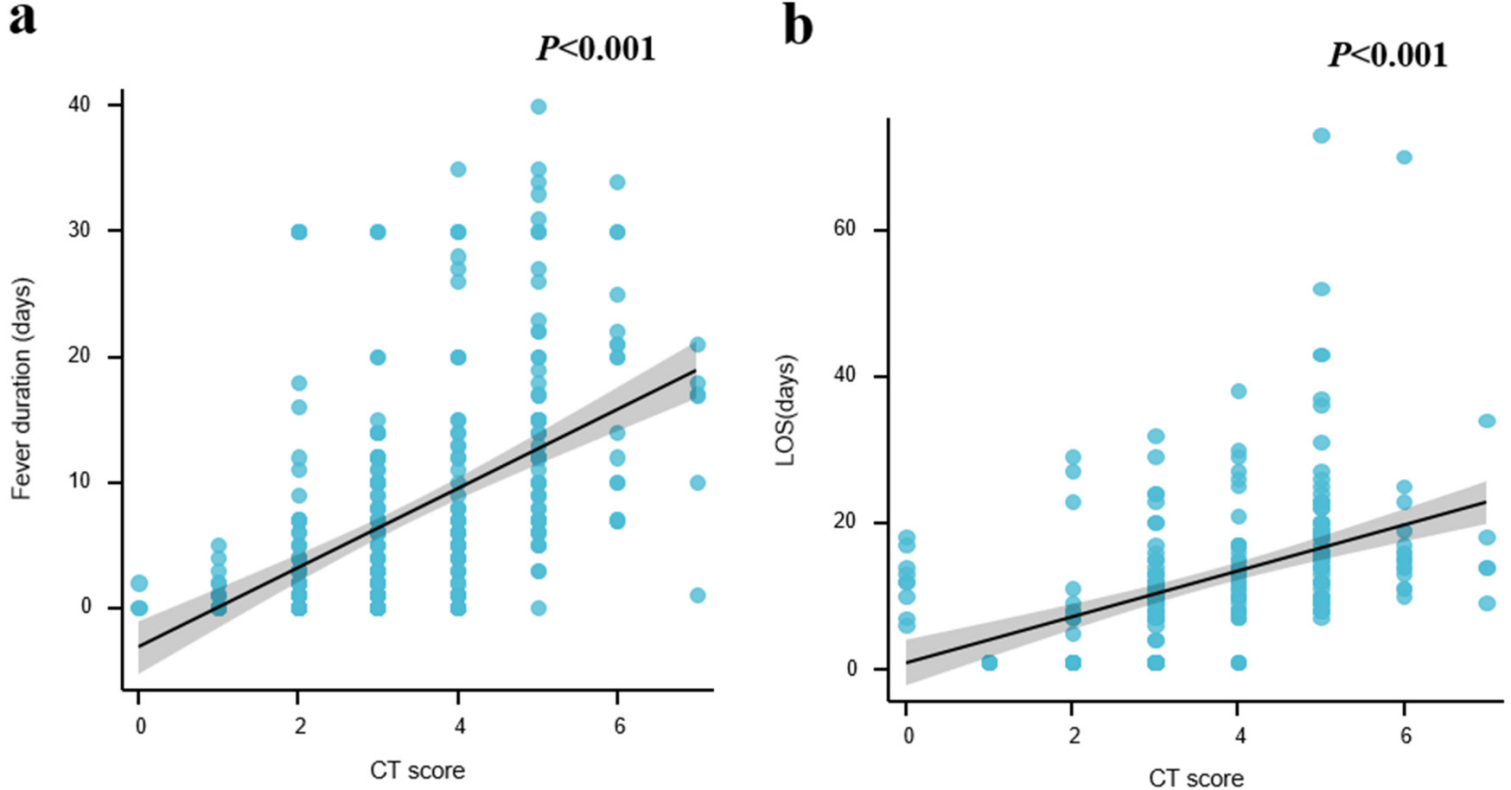
Comparison of semiquantitative chest CT score and prognosis. **(a)** Fever duration. **(b)** Length of stay (LOS). *P < 0.05; **P < 0.01; ***P < 0.001; ****P < 0.0001.

Since the chest CT score played an important role in the disease severity of CAP, we attempted to evaluate the prognosis according to chest CT score. With this scoring system, we found that chest CT scores positively correlated with fever duration and length of stay (LOS) (*P* < 0.001) (Figure 5). It was concluded that the chest CT score was valuable in estimating the course of diseases.

## 4 Discussion

Community-acquired pneumonia (CAP) is a common cause of outpatient visits and hospital admissions for children worldwide (16). While the usefulness of chest radiograph in diagnosing CAP accurately has been demonstrated (17, 18), computed tomography (CT) is an imaging tool typically reserved for children when complications are suspected or where there is difficulty in differentiating CAP from other pathology (17, 19). This limitation in the number of studies that include patients of the pediatric age group that use chest CT might be because CT imaging has been performed in a smaller number of cases, despite CAP having a higher incidence in pediatric cases. The novelty of our study is that we report our chest CT results in pediatric cases and assess the value of a semiquantitative chest CT scoring system in estimating the severity of pediatric CAP.

We measured the size of lung involvement proportionally to the thoracic cavity size and added the consolidation in a semiquantitative chest CT scoring system for comprehensive evaluation; this is an innovation in our study. In a previous study, the authors concluded that the size of consolidation and the side of its location could be used as predictors of severity of CAP among children aged >12 months (20). The study's scoring system evaluates lobar involvement and hydrothorax, which aligns with prior research demonstrating that extent of lung consolidation correlates with disease severity in pneumonia (17, 20). What’s important is that the CT scoring system is not correlated with age, weight, or gender, and the system has high consistency (ICC value of 0.957) among the observers. In other words, the system has feasibility for children.

Our study showed that chest CT resulted in a diagnosis of CAP in >90% of cases, and the upper lobes were the most frequently involved. The previous research pointed to the necessity of hospitalizing children with CAP aged from 1 year onward with upper lobe involvement (21); our results support the same view. The abnormal chest CT findings of pediatric patients with CAP are similar to those CT findings of adults; patchy shadows and consolidations were the most common. Moreover, the CT scoring system synthesized multiple CT performances according to the size of lung involvement for effective assessment in our study.

Numerous studies have shown that there is a good relationship between laboratory values and clinical condition in pediatric CAP patients (22, 23). Likewise, we found that chest CT scores had a positive correlation with NEU%, neutrophil count, and CRP, PCT, LDH, and FIB levels and had a negative correlation with lymphocyte count and HGB and ALB levels. These laboratory values have been proved to predict the severity of CAP (24–26). What’s interesting is that ALB is strongly correlated with score. As an indicator of nutritional status, serum ALB has been associated with the risk of progressive disease among patients with pneumonia (6, 27). Moreover, hypoalbuminemia (ALB < 30 g/L) could reduce plasma osmolality and enhance the exudation within the lungs, thus exacerbating the infection (28). Therefore, chest CT scores correlated strongly with systemic inflammatory biomarkers of CAP as a potential predictor of severe cases.

The relationship between chest CT scores and pathogens, on the other hand, showed that coinfection had a higher score than any single infection. In the previous reports, compared with children infected with a single pathogen, those with coinfections had a higher frequency of leukocytosis, consolidation on chest radiography, parapneumonic effusions, intensive care unit admission, need for mechanical ventilation, and an increased length of hospital stay (29). It is demonstrated that coinfection is likely to enhance the severity of CAP (7). Likewise, semiquantitative chest CT score has the same character. Regretfully, we did not specifically analyze the score of different coinfection types on account of limited cases. However, we could attempt to deduce coinfections from the image performance and chest CT score for guiding clinical treatment.

It is widely known that the size of lung involvement is directly related to the situation of the disease (20). Therefore, we analyzed the relationship between chest CT score and disease severity. According to ROC analysis of the CT score, its sensitivity and specificity in predicting severe CAP were high, and CAP was likely to develop a severe form when the score was higher than 3. Furthermore, chest CT score had a positive correlation with fever duration and LOS; it reconfirmed that chest CT score was closely related to disease condition. These findings suggested that semiquantitative chest CT score may help predict severity of the disease.

The study has several limitations. First, it was a retrospective, single-center study, which may introduce selection bias and limit the generalizability of the findings. Future prospective studies with standardized CT referral criteria and inclusion of non-CT cases are needed to validate these results and minimize bias. Additionally, the study population was from a tertiary referral center, which may include more severe or complex cases than primary care settings. Second, the sample size was relatively small, and there was no external validation of the CT scoring system. In the future, we will conduct an external validation study to confirm its applicability in diverse populations. Third, the use of CT in children should consider radiation exposure risks, and the cost and accessibility of CT imaging may limit its widespread use, especially in resource-limited areas. The use of CT scans in our study was not routine but was guided by the need to assess disease severity and complications in patients where clinical suspicion warranted further investigation. This approach is consistent with the understanding that CT can play a crucial role in assessing severity in pediatric CAP, especially in cases where standard clinical and radiographic evaluations may not provide sufficient information. Our study aimed to evaluate the utility of a semiquantitative chest CT scoring system in estimating severity and evaluating prognosis in pediatric CAP, which could provide a valuable supplement to clinical and laboratory evaluations.

In the context of technological advancements in medical imaging analysis, recent studies have demonstrated the potential of sophisticated diagnostic techniques in enhancing disease assessment. For instance, the application of confidence-driven dynamic spatiotemporal convolutional networks has shown promise in improving diagnostic accuracy for neurodegenerative diseases (30). Similarly, multimodal brain network fusion techniques have been utilized to develop intelligent diagnostic devices, offering new perspectives in medical diagnostics (31). Furthermore, the use of stacked topologically preserving dynamic brain networks has provided advanced methods for representation and classification in medical imaging (32). While these studies focus on different medical fields, they collectively highlight the growing importance of integrating advanced imaging analysis techniques into clinical practice. In the case of our semiquantitative chest CT scoring system, future research could explore the integration of such advanced technologies to further enhance its utility in monitoring disease progression and improving diagnostic outcomes in pediatric CAP patients.

In summary, this study shows that there is a significant relationship between semiquantitative chest CT score and pediatric CAP severity, coinfection, and laboratory findings in children. This suggests that the semiquantitative chest CT scoring system can be used to assess the severity of the disease and can play an important role in clinical practice. The scoring system can serve as a valuable tool for communication among healthcare professionals. By providing a standardized assessment of disease severity, it can facilitate discussions between radiologists, pediatricians, and intensivists, ensuring a more coordinated approach to patient care. Furthermore, the scoring system may contribute to clinical research by providing a consistent metric for evaluating the efficacy of treatments and comparing outcomes across different patient populations. However, there are several challenges to consider when applying the scoring system in clinical practice. One potential limitation is the accessibility and cost of CT imaging in some healthcare settings, particularly in resource-limited areas. The accurate interpretation of chest CT scans requires specialized training and expertise. Further studies with larger numbers of patients are still needed to determine the significance of chest CT scores in clinical practice in pediatric patients with CAP.

## Data Availability

The raw data supporting the conclusions of this article will be made available by the authors, without undue reservation.

## Supplementary material

Supplementary Figure S1 Comparison of semiquantitative chest CT score and demographic characteristics. **(a)** Age; **(b)** Weight; **(c)** Gender.
