# Supplementary figures and images for: Semiquantitative chest computed tomography scoring system to estimate severity in pediatric community-acquired pneumonia
Source: Front Pediatr. 2025 Aug 4;13:1556349. doi: 10.3389/fped.2025.1556349 (PMC12358383; doi:10.3389/fped.2025.1556349)

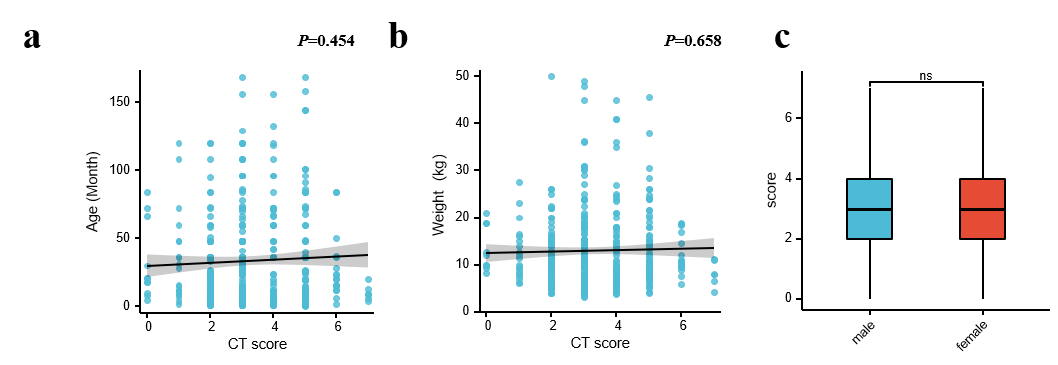

Supplement: Supplementary Figure S1 — Comparison of semiquantitative chest CT score and demographic characteristics. (a) Age; (b) Weight; (c) Gender. [file Image1.tif]
