# Supplementary material for: Semiquantitative chest computed tomography scoring system to estimate severity in pediatric community-acquired pneumonia
Source: Front Pediatr. 2025 Aug 4;13:1556349. doi: 10.3389/fped.2025.1556349 (PMC12358383; doi:10.3389/fped.2025.1556349)
Supplement: Supplementary file 2 [file Table1.docx]

| **Inter-rater agreement (kappa)** | |
| --- | --- |
| \| Observer A \| \| --- \| | Score1 |
| \| Observer B \| \| --- \| | Score2 |

| Score1 |
| --- |

| \| Score2 \| \| --- \| | \| 0 \| \| --- \| | \| 1 \| \| --- \| | \| 2 \| \| --- \| | \| 3 \| \| --- \| | \| 4 \| \| --- \| | \| 5 \| \| --- \| | \| 6 \| \| --- \| | \| 7 \| \| --- \| |  |
| --- | --- | --- | --- | --- | --- | --- | --- | --- | --- | --- | --- | --- | --- | --- | --- | --- | --- | --- |
| \| 0 \| \| --- \| | 11 | 0 | 0 | 0 | 0 | 0 | 0 | 0 | 11 (2.6%) |
| \| 1 \| \| --- \| | 0 | 20 | 0 | 0 | 0 | 0 | 0 | 0 | 20 (4.7%) |
| \| 2 \| \| --- \| | 0 | 0 | 92 | 4 | 0 | 0 | 0 | 0 | 96 (22.5%) |
| \| 3 \| \| --- \| | 0 | 0 | 2 | 135 | 4 | 0 | 0 | 0 | 141 (33.1%) |
| \| 4 \| \| --- \| | 0 | 0 | 0 | 9 | 60 | 0 | 0 | 0 | 69 (16.2%) |
| \| 5 \| \| --- \| | 0 | 0 | 0 | 1 | 9 | 56 | 0 | 0 | 66 (15.5%) |
| \| 6 \| \| --- \| | 0 | 0 | 0 | 0 | 0 | 15 | 2 | 0 | 17 (4.0%) |
| \| 7 \| \| --- \| | 0 | 0 | 0 | 0 | 0 | 4 | 2 | 0 | 6 (1.4%) |
|  | 11 | 20 | 94 | 149 | 73 | 75 | 4 | 0 | 426 |
|  | (2.6%) | (4.7%) | (22.1%) | (35.0%) | (17.1%) | (17.6%) | (0.9%) | (0.0%) |  |
| \| Weighted Kappaa \| \| --- \| | 0.95681 | |  |  |  |  |  |  |  |
| \| Standard error \| \| --- \| | 0.00676 | |  |  |  |  |  |  |  |
| \| 95% CI \| \| --- \| | 0.94356 to 0.97005 | |  |  |  |  |  |  |  |

a Quadratic weights
