# Supplementary material for: Semiquantitative chest computed tomography scoring system to estimate severity in pediatric community-acquired pneumonia
Source: Front Pediatr. 2025 Aug 4;13:1556349. doi: 10.3389/fped.2025.1556349 (PMC12358383; doi:10.3389/fped.2025.1556349)
Supplement: Supplementary file 3 [file Table2.docx]

| **eTable 2 CT findings in different pathogens** | | | | | |
| --- | --- | --- | --- | --- | --- |
| Radiology | Virus | Bacteria | Atypical pathogens | Coinfection | *P* value |
| Patchy shadows, No. (%) | 69(77.53) | 54(88.52) | 78(89.66) | 104(72.22) | 0.034 |
| Consolidation(>1 lobe), No. (%) | 31(34.83) | 15(24.59) | 36(41.38) | 68(47.22) | 0.0168 |
| Air bronchogram, No. (%) | 13(14.61) | 9(14.75) | 32(36.78) | 39(27.08) | 0.0014 |
| Hydrothorax, No. (%) | 0(0.00) | 18(29.51) | 15(17.24) | 27(18.75) | <0.0001 |
| Abscess, No. (%) | 0(0.00) | 6(9.84) | 1(1.15) | 9(6.25) | 0.0066 |
| Cavitation, No. (%) | 0(0.00) | 3(4.92) | 2(2.30) | 7(4.86) | 0.164 |
| Lymphadenopathy, No. (%) | 2(2.25) | 0(0.00) | 1(1.15) | 4(2.78) | 0.54 |
| Fibrotic lesions, No. (%) | 2(2.25) | 0(0.00) | 0(0.00) | 2(1.39) | 0.3982 |
| Nodules, No. (%) | 1(1.12) | 0(0.00) | 1(1.15) | 1(0.69) | 0.8555 |
